# Governing digital misinformation: A computational analysis of platform intervention using a three-party evolutionary game model

**DOI:** 10.1371/journal.pone.0351207

**Published:** 2026-07-17

**Authors:** Yaming Zhang, Xiaoyu Song

**Affiliations:** School of Economics and Management, Yanshan University, Qinhuangdao, Hebei, China; Shanghai Jiao Tong University, CHINA

## Abstract

The proliferation of misinformation on short video platforms poses a significant challenge to digital governance and public trust. While platforms are central actors, their strategic role in rumor governance remains underexplored. This study introduces a tripartite evolutionary game model that incorporates the platform as a bounded rational strategic actor alongside rumor spreaders and information clarifiers, and combines this model with the extended SICR (Susceptible-Rumor spreader-Clarifier-Recovered) information diffusion framework, where game strategies and payoffs are defined based on user roles. This combined framework allows us to analyze the strategic interactions among these three agents. Numerical simulations identify key evolutionary stable states and reveal that increasing the probability and perceived benefits of platform intervention can effectively steer the system towards an ideal equilibrium where platforms strictly supervise, and clarifiers actively debunk rumors. A case study of the “Hengshan Bus” incident supports the model’s applicability, demonstrating that proactive platform intervention accelerates the dissemination of corrective information. Our findings illustrate the important and strategically complex role of platforms in self-regulation, and provide insights for co-governance mechanism design on Chinese short-video platforms.

## 1. Introduction

A short video titled “Hunan Hengshan Bus Driver Fails to Brake on Curves” rapidly spread across the Douyin platform. On the day of its release, the video surpassed five million views, with the comment section flooded with condemnations such as “reckless driving,” “the driver should be taken to court,” and “I’ll never ride the bus again,” quickly propelling it to the top of trending topics. Notably, the platform did not block the rumor, nor did it issue an official response, allowing the falsehood to spread unchecked. It wasn’t until Douyin’s official account released a debunking video 48 hours later that public opinion shifted. The video clarified that the driver was utilizing inertia to navigate the curve, not neglecting safety measures. This time lag exposed governance issues within the platform: premature intervention risks unnecessary review costs due to misjudgment, while delayed action may trigger clarification crises once rumors become entrenched. The incident underscores the urgency of optimizing platform intervention strategies and provides a natural experimental setting for calibrating proposed three-party game models.

Current research on rumor governance has primarily followed two distinct exploratory pathways. The first involves modeling communication dynamics, as seen in the SIR (Susceptible-Infected-Recovered) model and its derivatives [1,2]. These models reveal general patterns of rumor propagation while reducing governance actors to exogenous intervention variables. The second pathway employs game theory to analyze inter-subject interactions. For example, Li et al. [3] developed a three-party game model involving the government, the media, and netizens to quantify strategic interactions. Additionally, Zhong et al. [4] proposed an intermittent control strategy to explore the trade-offs between regulatory costs and effectiveness. However, existing studies treat platforms as neutral information conduits and fail to account for their strategic evolution as independent stakeholders operating under information and cost constraints. There are two obvious research gaps in existing studies that limit in-depth analysis of short video platform rumor governance. First, social media platforms are merely regarded as neutral information transmission channels or exogenous regulatory variables; rather than as independent strategic actors with bounded rationality. The strategic evolution dilemma between their short-term commercial traffic benefits and long-term social responsibility and reputation gains has not been fully explored. Second, research on rumor diffusion dynamics and multi-subject game behavior is fragmented in the existing literature. There is a lack of integrated modeling methods that can reflect the dynamic process of information propagation and the strategic interaction of multiple participants in rumor governance simultaneously. To address these gaps, this study makes three innovative contributions. First, it takes the platform as an independent, bounded-rational strategic actor and constructs a tripartite evolutionary game model of platform-rumor spreader-clarifier. Second, it combines the extended SICR (Susceptible-Rumor spreader-Clarifier-Recovered) information diffusion model with evolutionary game theory to form a sequential combined framework of propagation dynamics and strategy evolution. Third, it verifies the theoretical model with real comment data from short video platforms to improve its external validity and practical guiding significance. The study aims to reveal the strategic interaction mechanism of multiple subjects in short video rumor governance and provide a clear theoretical basis for optimizing platform regulatory strategies and the constructing multi-party co-governance mechanisms.

The paper regards platforms as bounded rational strategic actors and constructs a three-party evolutionary game model involving platforms, rumor propagators, and information clarifiers. The core focus is the nonlinear trade-off between platform regulatory costs and rumor containment effectiveness. Focusing on the rumor clarification phase, the model uses replicator dynamics to depict the strategic evolution trajectory of platform as they navigate the high costs of early intervention and high risks of late intervention. Using the Hengshan incident as a case study, the model maps observable phenomena to key parameters. Rumor fermentation speed corresponds to propagation rate β; platform response time corresponds to intervention timing t₀; public sentiment subsidence speed to clarifier conversion rate γ. It should be noted that this study has clear applicability boundaries. It does not address initial rumor identification technologies; nor optimal control problems under fully rational assumptions. The objective of this paper is to provide a theoretical basis for selecting a platform intervention strategy under specific cost constraints; rather than pursuing a universal optimal solution.

This paper is structured as follows: Section 2 reviews relevant research and clarifies the study’s positioning; Section 3 introduces the model framework and fundamental assumptions; Section 4 constructs the three-party evolutionary game model and conducts stability analysis; Section 5 explores the impact of key parameters through numerical simulations; Section 6 performs parameter calibration and case validation using the Hengshan incident and Section 7 summarizes the study, outlining its limitations and future research directions.

## 2. Related research

In recent years, the widespread adoption of the internet and the advent of the 5G era have significantly expanded channels for information exchange. The real-time interactivity of online platforms has attracted massive user participation [5], thereby profoundly influencing public perception [6], viewpoints, and attitudinal tendencies. Simultaneously, the increasing prevalence of online rumors has introduced new challenges to social information governance. Against this backdrop, developing reasonable and effective platform intervention policies offers fresh approaches to rumor management. However, effectively controlling rumors requires not only technological measures but also a deep understanding of the mechanisms underlying the spread of misinformation. Consequently, current research on online rumors primarily focuses on two areas: the diffusion process of rumors and strategies for regulating them.

The SIR model has been widely employed in research on rumor propagation modeling. Scholars have proposed various extended models based on it to explain different characteristics of rumor transmission. These include the classic DK rumor propagation model [7], the SHIR model incorporating forgetting mechanisms [1], the dual-rumor parallel propagation model [2], the influence of user group size on rumor propagation [8], a game-theoretic analysis of public-private interactions in rumor propagation [9], and an analysis of rumor diffusion pathways based on super-spreading theory [10], constructing models for public opinion propagation and evolution under constrained conditions [11], and exploring the evolutionary patterns of online public opinion across temporal, geographical, and media usage dimensions [12].

In the study of online rumor evolution, researchers have introduced the SMQIR model [13], which shows that social media not only accelerates rumor spread but also extends its duration. Other studies have integrated epidemic models with intra-group delay effects to establish a dynamic delayed SEIR(Susceptible-Exposed-Infected-Recovered) evolutionary game model [14]. Further research has explored the application of blockchain technology in public opinion risk management models, using smart contracts to monitor and track public opinion trends [15].

In the domain of rumor governance, a variety of analytical models have been established. These include behavioral game models between online media and local governments, as well as regulatory mechanisms involving central government penalties for emergency crisis events [16]. Improved SEIR [17] and SEIQR [18] models have been applied to evaluate the scope and intensity of public opinion governance under government intervention. Using grey relational analysis and qualitative comparative analysis, some studies [19] have examined government public opinion governance from internal and external dimensions. Other studies have reclassified the roles in the SIR model into rumor spreaders and rumor debunkers, and constructed a SICR dual-game model to analyze the communication behaviors of short-video users [20]. Additional research has focused on media roles, such as proposing communication models with “reinforcement” and “dispersion” dimensions [21], introducing neutral groups to analyze the evolution of three types of public opinion under media influence [22], and evaluating the effects of joint media-government intervention on rumor control [23]. Existing studies have shown that government authority and media influence are critical to the effective dissemination of rumor-refuting information [24].

As important information carriers, social media platforms are responsible for content review, and the strictness of their supervision mechanisms is crucial to maintaining a healthy information ecosystem. Effective supervision can promptly identify and curb the spread of rumors and false information, protect the legitimate rights and interests of users, and maintain social stability. Moreover, platform-level supervision can positively guide user behavior and foster a positive online environment. However, as reflected in the above review, existing rumor governance research has mainly focused on the roles of media and government, while the role of platforms has received relatively insufficient attention. In recent years, evolutionary game theory has achieved remarkable methodological innovations in asymmetric interactions, social efficiency quantification, and spatial dynamic simulation [25,26]. Although these studies provide a solid theoretical foundation for exploring behavioral interactions and evolutionary stability, they are mostly applied to ecological evolution, energy systems, and other fields. Importantly, they have not been effectively applied to short-video rumor governance scenarios, especially by combining evolutionary game models with the SICR information diffusion framework and regarding platforms as independent, bounded-rational strategic actors.

In summary, despite fruitful achievements in rumor propagation models, evolutionary game theory, and platform governance, existing studies still have three major limitations. First, most studies focus on governments and media, while paying insufficient attention to platforms as independent strategic actors with the dual attributes of “commercial entity” and “governance subject”. Second, the SICR model and evolutionary game theory are often studied separately, lacking a combined framework suitable for short-video scenarios. Third, advanced evolutionary dynamics methods such as asymmetric interaction modeling and spatial simulation have not been applied to false information governance in short videos, leading to inadequate matching between research methods and specific scenarios.

Therefore, a critical research gap remains: the SICR-based rumor governance framework has not yet incorporated platforms as independent strategic actors, and there is a lack of organic integration between information diffusion dynamics and multi-party evolutionary games in the context of short-video platforms. This study aims to fill this core gap, which constitutes the starting point of theoretical innovation and methodological design in this paper.

In view of this, this study introduces a platform supervision mechanism into the classical SICR information dissemination model. We construct a tripartite evolutionary game model among platforms, rumor spreaders, and rumor clarifiers, to explore their dynamic interaction strategies and the impact of platform behavior on governance effects. Finally, we verify the model through a case study of the “Hengshan Bus” incident.

## 3. Problem description and assumptions

### 3.1. Introduce the SICR model of the platform body

**Fig 1:**
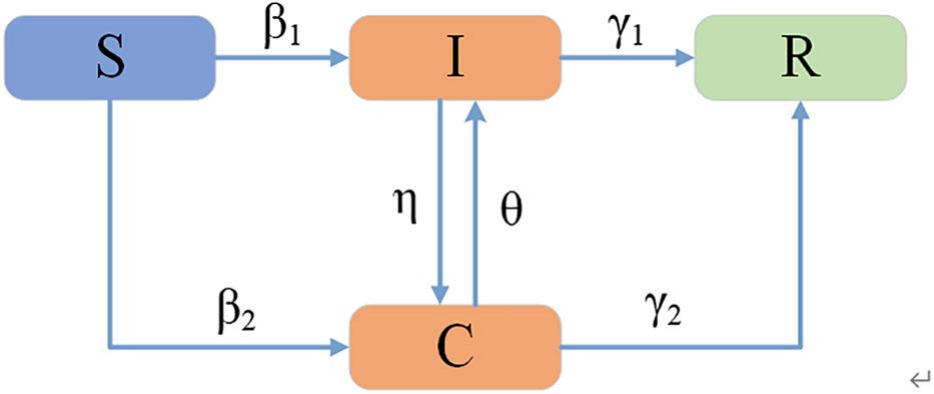
The propagation path of the SICR model.

The SICR model [20]extends the traditional SIR framework by distinguishing between two distinct types of propagators: rumor spreaders and information clarifiers. These groups exhibit mutual dependence and compete to influence others to adopt their respective viewpoints. This adversarial dynamic establishes a game-theoretic relationship between spreaders and clarifiers, which can accelerate rumor suppression and facilitate the restoration of truthful information. The propagation pathway of the SICR model is illustrated in Fig 1.

The arrows indicate transition pathways between population states, with parameters representing transition probabilities: β₁ (S → I), β₂ (S → C), γ₁ (I → R), γ₂ (C → R), η (I → C), θ (C → I). Individuals in state S are susceptible to influence from both rumor spreaders (I) and clarifiers (C). These two groups exhibit an adversarial game-theoretic relationship, each attempting to sway public opinion. Individuals in state R gradually lose interest in the discussion as the event evolves.

**Fig 2:**
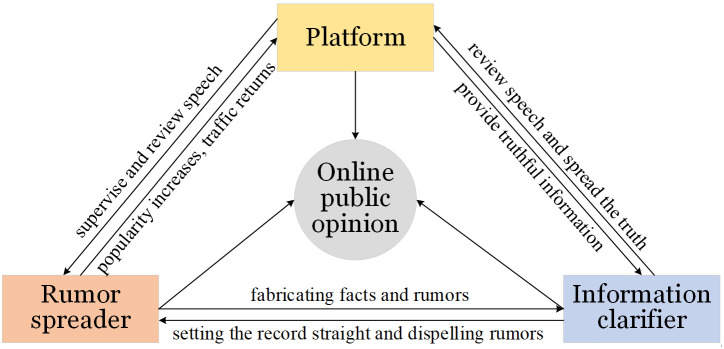
Platform-rumor spreader-clarifier tripartite game relationship diagram.

In the SICR model illustrated in Fig 1, individuals in state S—netizens unaware of relevant hot topics—are susceptible to influence from both rumor spreaders (I) and information clarifiers(C). These two groups exhibit an adversarial game-theoretic relationship, each attempting to sway public opinion by disseminating information and increasing their visibility, with the goal of altering the opposing party’s stance. Meanwhile, individuals in state R represent those who gradually lose interest in the discussion as the event evolves. The SICR propagation model underscores the critical role of clarifiers in countering rumors and persuading the public of the truth through proactive communication. Beyond clarifiers, government agencies and platforms also play active roles in the actual information dissemination process. Recognizing the importance of platform intervention in rumor refutation, this study incorporates the platform as a strategic actor into the game between rumor spreaders and clarifiers. The resulting tripartite game structure is depicted in Fig 2.

This diagram and Fig 1 form a consistent whole, Fig 1 defines the rules governing user state transitions, while this diagram focuses on the strategic interactions among key actors after transitions. Platform regulation influences the state transition probabilities depicted in Fig 1, and conversely, the behaviors of disseminators and clarifiers also impact platform decisions.

As a central hub for information dissemination, the platform bears the responsibility to analyze and monitor user-submitted content. However, it may also opt to forgo rigorous review and supervision of information providers, particularly when their content generates significant controversy or engagement. To mitigate the spread of misinformation, platforms employ content filtering and monitoring mechanisms for short videos, promote authentic and reliable information, and steer user attention toward authoritative sources. Through the implementation of content review policies and algorithm-driven recommendation systems, they reduce the visibility of rumors and effectively suppress their dissemination. A dynamic interaction exists among platforms, rumor spreaders, and clarifiers, making platform regulation a critical component in combating online rumors. Platforms prioritizing traffic and engagement may relax oversight, inadvertently facilitating rumor propagation. Conversely, those committed to maintaining a healthy online environment will enforce stricter regulations and amplify the voice of clarifiers.

Notably, participants in this three-party game exhibit characteristics distinct from traditional models, aligning closely with the attention economy and user-participatory governance attributes of short-video platforms:

Platform Operators: In this context, they function not only as information regulators but also as strategic participants with commercial interests. Their utility function encompasses both short-term traffic revenue (S₁) and long-term reputation gains (R_p_, S₂), with the primary challenge lying in balancing monetization of traffic with social responsibility. This manifests in their strategic choices {strict regulation z, lenient regulation 1-z} and the trade-off between audit costs (P₃) and regulatory benefits (S₂).

Rumor spreaders: Unlike passively “infected” individuals in SIR models, they are rational actors actively pursuing traffic gains. Their core motivation is to obtain clicks and credibility through controversial content (parameters M₁, M₂), while bearing fabrication costs such as editing and planning (I₁). Their strategy is dually influenced by platform regulation intensity (z) and clarifier behavior (y), forming dynamic evolutionary trajectories that fit short-video platforms’ traffic-driven ecosystem.

Information clarifiers: Possessing a dual identity as “users and supervisors,” they differ from passive “recovered” groups in traditional models. Their decisions balance debunking costs (C₁) and social recognition gains (R₁), as well as opportunity costs from the “spiral of silence” (C₂). Parameter aC_p_ captures the collaborative mechanism between platforms and clarifiers—platform intervention amplifies clarifiers’ voices and reduces their debunking costs, embodying the “official-citizen collaborative debunking” feature of short-video platforms.

There exists a two-way dynamic interaction mechanism among the three subjects in false information governance: the platform’s supervision strategy (z) and intervention probability (a) directly adjust the cost-benefit structure of rumor spreaders and clarifiers by changing their traffic gain, debunking cost and benefit; in turn, the rumor spreader’s propagation intensity (x) and clarifier’s clarification behavior (y) jointly determine the platform’s supervision cost ($P_{\text{3}}$) and reputation gain ($R_{P}$), forming a feedback loop of strategic interaction and parameter interdependence. This inter-subject interaction is the core logic of constructing the tripartite evolutionary game model, and all interaction effects are embedded into the payoff matrix and replication dynamic equations through parameter interaction terms (e.g., ${\text{a}C}_{P}$, xz, yz).

The proposed model follows a sequential combined structure. The SICR model defines user states and provides role settings for the evolutionary game, while the evolutionary game analyzes strategic interactions. The two modules work in logical order rather than as a fully coupled dynamic system.

### 3.2. Assumption proposed

Assumption 1: Social media platforms, rumor spreaders, and clarifiers are all bounded rationality.

Assumption 2: The strategy space for rumor spreaders is {spread rumors, not spread rumors}. The probability of choosing to spread rumors at time *t* is x (t) (abbreviated as x,0≤x≤1), and the probability of choosing not to spread rumors is 1−x; The strategy space of the clarifier is {clarify, not clarify}. The probability of choosing to clarify at time *t* is  y (t) (abbreviated as y, 0 ≤ y ≤ 1), and the probability of choosing not to clarify is 1−y; The regulatory behavior strategy of the platform is {strict supervision, loose supervision}. The probability of choosing strict supervision at time *t* is z (t), (abbreviated as z, 0 ≤ z ≤ 1), and the probability of choosing loose supervision is 1−z. Strict supervision refers to verifying false information on platforms and striving to restore a healthy and positive online environment; Loose supervision refers to the platform’s tacitly allowing netizens participating in event discussions to spread false information and pollute the online environment in order to gain attention and popularity. The aforementioned “strict oversight” is defined by measures such as content moderation and algorithmic throttling, aimed at suppressing the propagation of rumors and amplifying clarifications, thereby reflecting its social responsibility attributes. Conversely, “lax oversight” signifies a deliberate decision to permit rumor propagation for the purpose of short-term traffic enhancement, thus unveiling its commercial entity nature. The “spreading rumors” strategy employed by rumor propagators is centered on monetizing traffic, while the “clarifying” strategy of fact-checkers is aimed at balancing personal reputation with public interest. The three strategic choices under consideration align closely with their distinct characteristics.

Assumption 3: The platform regulates both rumor spreaders and clarifiers, and all three parties follow the goal of maximizing their own interests.

Assumption 4: All parameters are non-negative real numbers. To reflect the realistic bounded rationality of each party in the context of short video platforms, the following basic relationships among parameters are assumed: For rumor spreaders, the long-term credibility benefit (M₂) is greater than the short-term traffic benefit (M₁), and both exceed the cost of fabricating rumors (I₁), i.e., M₂ > M₁ > I₁ > 0. This ensures that their strategy choice involves a trade-off between immediate and future gains. For clarifiers, the opportunity cost of silence (C₂) exceeds the cost of active clarification (C₁), i.e., C₂ > C₁ > 0. This reflects the “spiral of silence” logic, where the cost of inaction is higher. Furthermore, the benefit of collaborative clarification (R₂) is greater than that of independent clarification (R₁), i.e., R₂ > R₁ > 0. For the platform, the audit cost (P₃) is positive (P₃ > 0). The intervention probability (a) is a bounded rational value between 0 and 1 (0 < a < 1), reflecting the uncertainty in real-world decision-making. All parameters are defined on a common ordinal scale based on the relative inequalities in Assumption 4. Their absolute magnitudes do not correspond to empirically measurable quantities. Based on the above assumptions, provide the payoff matrix for the platform, rumor spreader, and clarifier, as shown in Table 1.

**Table 1:** Payoff matrix of the platform, rumor spreader, and clarifier under strict and loose supervision.

Platform reviews the published information (strictly supervise *z*)			
Clarifier	Rumor spreader		
	Clarify *y*	Spread rumor *x*	Not spread rumor 1−x
		$S_{\text{2}}-aC_{P}+R_{p}-P_{\text{3}}$	$R_{P}-P_{\text{3}}-aC_{P}$
		$M_{\text{1}}-I_{\text{1}}$	*0*
		$aC_{P}+R_{\text{2}}-C_{\text{1}}$	$R_{\text{2}}-C_{\text{1}}+aC_{P}$
	Not clarify 1−y	$S_{\text{2}}-aC_{P}+R_{P}-P_{\text{3}}$	$-P_{\text{3}}$
		$M_{\text{2}}-I_{\text{1}}$	0
		$-C_{\text{2}}+R_{\text{1}}$	0
**Platform reviews the published information (loosely supervise 1−z)**			
Clarifier	Rumor spreader		
	Clarify *y*	Spread rumor *x*	Not spread rumor 1−x
		$S_{\text{1}}$	$S_{\text{1}}$
		$M_{\text{1}}-I_{\text{1}}$	0
		$R_{\text{1}}-C_{\text{1}}$	$-C_{\text{1}}$
	Not clarify 1−y	$S_{\text{1}}$	0
		$M_{\text{2}}-I_{\text{1}}$	0
		$R_{\text{1}}-C_{\text{2}}$	$-C_{\text{2}}$

The correspondence between parameter definitions and main characteristics is shown in Table 2. All parameters in this table are set with explicit justification to ensure the model configuration’s validity: ①Literature-based parameters: Core parameters such as platform verification cost$C_{P}$ rumor creation cost$I_{\text{1}}$, and platform revenue $S_{\text{1}}\;S_{\text{2}}\;$ reference [3,13,20], and other seminal studies on rumor governance and evolutionary games, ensuring parameter magnitudes align with academic conventions; ② Real-world logic: The opportunity cost of silence ($C_{\text{2}}$) is grounded in the “spiral of silence” theory. Public trust benefits ($R_{p}$) and collaborative debunking benefits ($R_{\text{2}}$) align with short-video platforms’ “attention economy” and “government-citizen collaboration” characteristics, with explicit relative parameter relationships (e.g., $C_{\text{2}}$ > $C_{\text{1}}$, $R_{\text{2}}$> $R_{\text{1}}$); ③ Empirical Calibration: Platform intervention probability a and initial states S/I/C/R are calibrated using data from the “Hengshan Bus” incident. Key parameters undergo sensitivity analysis to validate robust conclusions.

**Table 2:** Definition of each party’s parameters.

Party	Parameter	Meaning	Corresponding to the unique characteristics of the subject
Platform	$S_{\text{1}}$	Benefits of introducing loose regulations on the platform.	Commercially driven (short-term monetization of traffic)
	$S_{\text{2}}$	The platform takes the lead in strict supervision and eradication of misinformation resulting in a healthier virtual space.	Social Responsibility (Promoting a Clean and Healthy Online Environment)
	$C_{P}$	The costs of reviewing when platforms impose strict regulations.	Regulatory cost constraints
	$P_{\text{3}}$	The costs of the platform’s ongoing event data collection and audit rule formulation	Regulatory cost constraints
	$R_{P}$	The public begins to trust the platform, which is closely monitored.	Long-term Reputation Capital
	a	The likelihood that the clarifier will take the lead in screening the information represents the platform’s intervention probability, which is influenced by the clarifier’s proactive screening.	Cooperative Mechanism with Clarifiers
Rumor spreader	$M_{\text{1}}$	When the platform has inadequate oversight, the traffic that the rumor spreader receives.	Traffic-driven motivation (short-term popularity gains)
	$M_{\text{2}}$	The clarifier of information loses his voice, and the rumor spreader gains credibility.	Traffic-Driven Motivation
	$I_{\text{1}}$	The cost of time for rumor mongers to fabricate false information.	Production costs of actively spreading rumors
Clarifier	$C_{\text{1}}$	The cost of clarifiers in finding the truth and gathering truthful information.	Cost Constraints of Debunking Misinformation
	$C_{\text{2}}$	The loss that clarifiers delay speaking out, and are coerced by rumors and lose the right to speak.	The Opportunity Cost of Silence
	$R_{\text{1}}$	Intrinsic social recognition and informal reputation gain obtained by clarifiers from independent rumor debunking without platform intervention.	Fact-Checking Incentives (Basic non-material social recognition for spontaneous debunking behavior driven by social responsibility)
	$R_{\text{2}}$	The clarifier and the platform work together to clean up rumors and clarify the facts.	Synergies with the platform

### 3.3. Ethical approval and informed consent

This study utilized publicly available data from the Douyin platform. All data were anonymized and aggregated prior to analysis. No personally identifiable information was collected or used. As such, ethical approval and informed consent were not required for this research.

## 4. Model construction and stability analysis

### 4.1. Model building

Platforms, rumor spreaders, and clarifiers all begin the process of disseminating public opinion from their own interests. They then select strategies based on the maximization of benefits. Finally, they continuously modify their strategies as public opinion events unfold, eventually arriving at a stable final strategy through a series of adjustments.

(1) Platform strategy evolution analysis

If the expected return of platform intervention is $U_{\text{1}}$, the expected return of non-intervention is $\;U_{\text{2}}$, and the average expected return is $U_{\text{3}}$, then the calculation method is shown in Equations (1)–(3).


$$\begin{array}{c} U_{\text{1}}=x(y-\text{1})\left(P_{\text{3}}-S_{\text{2}}-R_{P}+aC_{P}\right)+(\text{1}-x)y\left(R_{P}-P_{\text{3}}-{aC}_{P}\right)+x(\text{1}-y)\left(S_{\text{2}}-{aC}_{P}+R_{P}-P_{\text{3}}\right)+(\text{1}-x)(\text{1}-y)\left(-P_{\text{3}}\right) \end{array}$$ (1)

$$\begin{array}{c} U_{\text{2}}=xy\left(S_{\text{1}}\right)+(\text{1}-x)y\left(S_{\text{1}}\right)+x(\text{1}-y)\left(S_{\text{1}}\right) \end{array}$$ (2)

$$\begin{array}{cc} U_{\text{3}}= & \;(z\;-\;\text{1})*\left(S_{\text{1}}x(y-\text{1})+S_{\text{1}}y(x-\text{1})-S_{\text{1}}xy\right)+z(x(y-\text{1})\left(P_{\text{3}}-R_{P}-S_{\text{2}}+aC_{P}\right)\\ & -P_{\text{3}}(x-\text{1})(y-\text{1})+y(x-\text{1})\left(P_{\text{3}}-R_{P}-aC_{P}\right)-xy(P_{\text{3}}-R_{P}-S_{\text{2}}+aC_{P}) \end{array}$$ (3)

The replication dynamic equation for the platform is shown in Equation (4). Platform replication dynamic Equation (4) includes interaction terms for x (rumor spreader strategy) and y (clarifier strategy), precisely because platform regulatory decisions must dynamically adjust based on the behaviors of the other two parties, reflecting its “decision dilemma under limited information” characteristic.


$$\begin{array}{c} Fz=z*(z\;-\;\text{1})*\left(P_{\text{3}}\;-xR_{P}+S_{\text{1}}x-S_{\text{2}}x-R_{P}y+S_{\text{1}}y+aC_{P}x+aC_{P}y+xyR_{P}-xyS_{\text{1}}-xyaC_{P}\right) \end{array}$$ (4)

(2) An Analysis of the Evolution of Rumor Spreaders’ Strategies

If the expected return of the rumor spreader is $A_{\text{1}}$, the expected return of non-rumor spreading is $A_{\text{2}}$, and the average expected return is $A_{\text{3}}$, then the calculation method is shown in Equations (5)–(7).


$$\begin{array}{c} A_{\text{1}}=yz\left(M_{\text{1}}-I_{\text{1}}\right)+z(\text{1}-y)\left(M_{\text{2}}-I_{\text{1}}\right)+y(\text{1}-z)\left(M_{\text{1}}-I_{\text{1}}\right)+(\text{1}-y)(\text{1}-z)\left(M_{\text{2}}-I_{\text{1}}\right) \end{array}$$ (5)

$$\begin{array}{c} A_{\text{2}}=\text{0} \end{array}$$ (6)

$$\begin{array}{c} A_{\text{3}}=-x\left(I_{\text{1}}-M_{\text{2}}-M_{\text{1}}y+M_{\text{2}}y\right) \end{array}$$ (7)

The replication dynamic equation for the rumor spreader is shown in Equation (8). The comparison of M₁, M₂, and I₁ in the rumor-spreader Equation (8) directly reflects their “benefit-cost” decision logic, consistent with the “proactive traffic-driven” characteristic.


$$\begin{array}{c} Fx=x(x-\text{1})\left(I_{\text{1}}-M_{\text{2}}-M_{\text{1}}y+M_{\text{2}}y\right) \end{array}$$ (8)

It should be noted that the platform strategy z does not appear explicitly in Equation (8). This algebraic independence arises from simplification but also reveals a structural limitation of the current payoff design, rather than merely a mathematical coincidence. Although z enters the expected payoff function of rumor spreaders, it does not appear explicitly in their replicator dynamics. This indicates that platform intervention influences spreader behavior indirectly through system equilibrium conditions, rather than directly affecting their immediate strategy updates. This constraint reflects the scope of the present model and suggests that alternative payoff structures could be explored in future work to establish a direct dependence between spreader dynamics and platform intervention.

(3) Evolutionary analysis of clarifier strategies

If the expected return of the clarifier is $B_{\text{1}}$, the expected return of the non-clarified information is $B_{\text{2}}$, and the average expected return is $B_{\text{3}}$, then the calculation method is shown in Equations (9)–(11).


$$\begin{array}{c} B_{\text{1}}=xz\left(R_{\text{2}}-C_{\text{1}}+aC_{P}\right)+(\text{1}-x)z\left(R_{\text{2}}-C_{\text{1}}+aC_{P}\right)+x(\text{1}-z)\left(R_{\text{1}}-C_{\text{1}}\right)+(\text{1}-x)(\text{1}-z)\left(-C_{\text{1}}\right) \end{array}$$ (9)

$$\begin{array}{c} B_{\text{2}}=xz\left(R_{\text{1}}-C_{\text{2}}\right)+x(\text{1}-z)\left(R_{\text{1}}-C_{\text{2}}\right)+(\text{1}-x)(\text{1}-z)\left(-C_{\text{2}}\right) \end{array}$$ (10)

$$\begin{array}{cc} B_{\text{3}}=\; & (y\;-\;\text{1})\left(C_{\text{2}}(x-\text{1})(z-\text{1})-x\left(C_{\text{2}}-R_{\text{1}}\right)(z-\text{1})+xz\left(C_{\text{2}}-R_{\text{1}}\right)\right)\\ & +y\left(x\left(C_{\text{1}}-R_{\text{1}}\right)(z-\text{1})+xz\left(R_{\text{2}}-C_{\text{1}}+aC_{P}\right)-C_{\text{1}}(x-\text{1})(z-\text{1})-z(x-\text{1})\left(R_{\text{2}}-C_{\text{1}}+aC_{P}\right)\right) \end{array}$$ (11)

The replication dynamic equation for the clarifier is shown in Equation (12). The introduction of the aC_p_ term in the clarifier Equation (12) mathematically captures the “platform-clarifier synergy” characteristic, distinguishing it from traditional models that assume clarifiers act in isolation.


$$\begin{array}{c} F_{y}=-y(y\;-\;\text{1})\left(C_{\text{2}}-C_{\text{1}}-C_{\text{2}}z+R_{\text{2}}z+aC_{P}z+xzC_{\text{2}}-xzR_{\text{1}}\right) \end{array}$$ (12)

### 4.2. Equilibrium and stability analysis

According to Friedman’s [27] method, the evolutionary stability strategy(ESS) of differential equation systems can be obtained by analyzing the local stability of the Jacobian matrix of the system. According to formulas(4), (8), and(12), the Jacobian matrix of the system can be obtained, as shown in formula(13).


$$\begin{array}{c} A=[\begin{array}{ccc} (\text{2}x-\text{1})\lambda_{\text{3}} & x(\text{1}-x)\left(M_{\text{1}}-M_{\text{2}}\right) & \text{0}\\ y(\text{1}-y)\left(C_{\text{2}}z-R_{\text{1}}z\right) & (\text{1}-\text{2}y)\lambda_{\text{2}} & y(\text{1}-y)\left(R_{\text{2}}-C_{\text{2}}+aC_{P}+C_{\text{2}}x-R_{\text{1}}x\right)\\ z(\text{1}-z)\lambda_{\text{4}} & z(\text{1}-z)\lambda_{\text{5}} & (\text{2}z-\text{1})\lambda_{\text{1}} \end{array}] \end{array}$$ (13)

Thereinto:


$$\begin{array}{c} \lambda_{\text{1}}=P_{\text{3}}-R_{P}x+S_{\text{1}}x-S_{\text{2}}x-R_{P}y+S_{\text{1}}y+aC_{P}x+aC_{P}y+R_{P}xy-S_{\text{1}}xy-aC_{P}xy \end{array}$$

$$\begin{array}{c} \lambda_{\text{2}}=C_{\text{2}}-C_{\text{1}}-C_{\text{2}}z+R_{\text{2}}z+aC_{P}z+C_{\text{2}}xz-R_{\text{1}}xz \end{array}$$

$$\begin{array}{c} \lambda_{\text{3}}=I_{\text{1}}-M_{\text{2}}-M_{\text{1}}y+M_{\text{2}}y \end{array}$$

$$\begin{array}{c} \lambda_{\text{4}}=R_{P}-S_{\text{1}}+S_{\text{2}}-aC_{P}-R_{P}y+S_{\text{1}}y+aC_{P}y \end{array}$$

$$\begin{array}{c} \lambda_{\text{5}}=R_{P}-S_{\text{1}}-aC_{P}-R_{P}x+S_{\text{1}}x+aC_{P}x \end{array}$$

Let F(x)=F(y)=F(z)=0, the local equilibrium points can be obtained as $E_{\text{1}}(\text{0},\text{0},\text{0})$, $E_{\text{2}}(\text{0},\text{0},\text{1})$, $E_{\text{3}}(\text{0},\text{1},\text{0})$, $E_{\text{4}}(\text{0},\text{1},\text{1})$, $E_{\text{5}}(\text{1},\text{0},\text{0})$, $E_{\text{6}}(\text{1},\text{0},\text{1})$, $E_{\text{7}}(\text{1},\text{1},\text{0})$, $E_{\text{8}}(\text{1},\text{1},\text{1})$. The above eight equilibrium points are substituted into the Jacobian matrix, and the eigenvalues of the Jacobian matrix corresponding to the equilibrium points are obtained as shown in Table 3.

**Table 3:** Eigenvalues of Jacobian matrices.

The eigenvalue of the game equilibrium solution			
Equilibrium point	Eigenvalue $I_{\theta }$	Eigenvalue $C_{\theta }$	Eigenvalue $P_{\theta }$
$E_{1}\left(0,0,0\right)$	$M_{\text{2}}-I_{\text{1}}$	$C_{\text{2}}-C_{\text{1}}$	$-P_{\text{3}}$
$E_{2}\left(0,0,1\right)$	$M_{\text{2}}-I_{\text{1}}$	$R_{\text{2}}-C_{\text{1}}+aC_{P}$	$P_{\text{3}}$
$E_{3}\left(0,1,0\right)$	$M_{\text{1}}-I_{\text{1}}$	$C_{\text{1}}-C_{\text{2}}$	$R_{P}-P_{\text{3}}-aC_{P}-S_{\text{1}}$
$E_{4}\left(0,1,1\right)$	$M_{\text{1}}-I_{\text{1}}$	$C_{\text{1}}-R_{\text{2}}-aC_{P}$	$P_{\text{3}}-R_{P}+S_{\text{1}}+aC_{P}$
$E_{5}\left(1,0,0\right)$	$I_{\text{1}}-M_{\text{2}}$	$C_{\text{2}}-C_{\text{1}}$	$R_{P}-P_{\text{3}}-aC_{P}-S_{\text{1}}+S_{\text{2}}$
$E_{6}\left(1,0,1\right)$	$I_{\text{1}}-M_{\text{2}}$	$C_{\text{2}}-C_{\text{1}}-R_{\text{1}}+R_{\text{2}}+aC_{P}$	$P_{\text{3}}-R_{P}+aC_{P}+S_{\text{1}}-S_{\text{2}}$
$E_{7}\left(1,1,0\right)$	$I_{\text{1}}-M_{\text{1}}$	$C_{\text{1}}-C_{\text{2}}$	$R_{P}-P_{\text{3}}-aC_{P}-S_{\text{1}}+S_{\text{2}}$
$E_{8}\left(1,1,1\right)$	$I_{\text{1}}-M_{\text{1}}$	$C_{\text{1}}-C_{\text{2}}+R_{\text{1}}-R_{\text{2}}$	$P_{\text{3}}-R_{P}+aC_{P}+S_{\text{1}}-S_{\text{2}}$

When $\;I_{\theta }\leq \text{0}$ and $\;C_{\theta }\leq \text{0}$ and $\;P_{\theta }\leq \text{0}$, the corresponding equilibrium point is the evolutionary strategy stability point. As can be seen from Table 3, the eigenvalues of $E_{\text{2}}(\text{0},\text{0},\text{1})$, $E_{\text{4}}(\text{0},\text{1},\text{1})$ and $E_{\text{6}}(\text{1},\text{0},\text{1})$ are all greater than 0, so they are not stable points of evolutionary strategy. The remaining equilibrium points are analyzed, and the following conclusions are drawn*.*

For $E_{\text{4}}\;$ (0,1,1), the clarifier eigenvalue $C_{\theta }=R_{\text{2}}-C_{\text{1}}+aC_{p}>\text{0}$. This is because the collaborative debunking benefit of clarifiers with platform synergy ($R_{\text{2}}+aC_{p}$) is always greater than the debunking cost ($C_{\text{1}}$) in the actual rumor governance scenario, leading to a positive $C_{\theta }$ that violates the ESS stability condition.

For $E_{\text{6}}\;$ (1,0,1), the clarifier eigenvalue $C_{\theta }=C_{\text{2}}-C_{\text{1}}-R_{\text{1}}+R_{\text{2}}+aC_{p}>\text{0}$. The positive value is determined by the core parameter relationship of clarifiers ($R_{\text{2}}+aC_{p}$ > $R_{\text{1}}+C_{\text{1}}-C_{\text{2}}$) and the realistic setting of collaborative debunking benefit, which makes the eigenvalue unable to be negative and thus the point is unstable.

In summary, $E_{\text{4}}\;$ and $E_{\text{6}}$ have at least one positive eigenvalue of the Jacobian matrix, which does not meet the necessary and sufficient condition for ESS, so they are not evolutionary stable points of the tripartite game system.

**Conclusion 1.** When $I_{\text{1}}>M_{\text{2}}$, $C_{\text{1}}>C_{\text{2}}$, $P_{\text{3}}>\text{0}$, the eigenvalues corresponding to the equilibrium point $E_{\text{1}}(\text{0},\text{0},\text{0})$ are all less than 0, and the strategy combination at this time is (not spread rumor, not clarify, loose supervision). The clarifiers decide not to clarify because the losses resulting from the rumors are less than the cost of discovering the truth. The cost of reviewing the information on the platform at this time is bound to be greater than 0, which is eternally established. In this scenario, the cost of fabricating rumors is greater than the trust gain obtained when creating rumors, so they tend not to spread rumors.

**Conclusion 2.** When $I_{\text{1}}>M_{\text{1}}$, $C_{\text{2}}>C_{\text{1}}$, $R_{P}<P_{\text{3}}+aC_{P}+S_{\text{1}}$, the eigenvalues corresponding to the equilibrium point $E_{\text{3}}(\text{0},\text{1},\text{0})$ are all less than 0, and the strategy combination is (not spread rumor, clarify, loose supervision). There are several reasons why the platform’s implementation of strict supervision is not as beneficial as it could be. For example, the cost of the rumor-monger is greater than the gain of attention when the rumor-monger is spreading, so they choose not to spread the rumor; the loss caused by the clarifier being coerced by the rumor is higher than the cost of finding the truth, so they will speak out to clarify; and finally, the platform’s decision to relax supervision is motivated by their own considerations.

**Conclusion 3.** When $M_{\text{2}}>I_{\text{1}}$, $C_{\text{1}}>C_{\text{2}}$, $P_{\text{3}}+aC_{P}+S_{\text{1}}>R_{P}+S_{\text{2}}$, the equilibrium point $E_{\text{5}}(\text{1},\text{0},\text{0})$ corresponds to the eigenvalues are less than 0, and the strategy combination is (spread rumor, not clarify, loose supervision). Currently, the benefits of public trust that rumor-mongers receive for taking the initiative to speak out outweigh the costs incurred in spreading the rumors, so they decide to do so; the costs associated with restoring the truth outweigh the loss of the rumor environment, so they decide not to provide clarification; the platform will adopt relaxed supervision because the benefits of strict online order maintenance and public trust gained from strict supervision add up to less than the costs associated with reviewing information, verifying information to dispel rumors, and assessing information.

**Conclusion 4.** When $M_{\text{1}}>I_{\text{1}}$, $C_{\text{2}}>C_{\text{1}}$, $P_{\text{3}}+aC_{P}+S_{\text{1}}>R_{P}+S_{\text{2}}$, the equilibrium point $E_{\text{7}}(\text{1},\text{1},\text{0})$ corresponds to the eigenvalues are less than 0, and the strategy combination is (spread rumor, clarify, loose supervision). Currently, the benefits of the discussion outweigh the costs incurred by the rumor-mongers, and the benefits of clarifying information to counter rumors outweigh the losses caused by the rumors and the strict maintenance of online order. Moreover, the benefits of public trust gained through the platform’s strict supervision and the benefits of strictly upholding online order are less than the benefits of reviewing information, verifying information, and dispelling rumors, as well as the benefits of lax supervision.

**Conclusion 5.** When $M_{\text{1}}>I_{\text{1}}$, $R_{\text{2}}+C_{\text{2}}>C_{\text{1}}+R_{\text{1}}$, $P_{\text{3}}+aC_{P}+S_{\text{1}}<R_{P}+S_{\text{2}}$, the equilibrium point $E_{\text{8}}(\text{1},\text{1},\text{1})$ are all less than 0, and the strategy combination is (spread rumor, clarify, strict supervision). In this case, the rumor-monger’s benefit from spreading rumors to attract followers exceeds the cost of rumor-mongering, so the clarifier will choose to spread rumors; the total information benefits from the rumors’ losses and the platform’s assistance in suppressing rumors and upholding the online environment exceed the total costs of gathering accurate information and the benefits of public attention when silence; the clarifiers will opt to speak up and challenge the rumors; and the total benefits of public trust gained from the platform’s strict oversight and the advantages of strictly maintaining network order exceed the cost of information review. Given the advantages of lax oversight compared to the expense of fact-checking and dispelling myths, the platform will, in its own best interests, decide to uphold online order and employ closely monitored.

In summary, the strategies presented by $\;E_{\text{7}}\;$ (1,1,0) and $E_{\text{8}}\;$ (1,1,1) are closer to the real dissemination of online public opinion. This is due to their conformity to the phased evolution of short-video platform rumor governance (early lax supervision for traffic, late strict supervision for reputation) and the real feature of coexistent rumor spreading and spontaneous clarification, a conclusion derived from the model’s stability analysis and realistic parameter setting. The platform, as a part of the regulatory authorities, sometimes chooses to relax supervision for the sake of traffic, eyeballs, and discussion heat, but as long as the benefits of a good network environment brought by strict supervision are greater than the heat benefits of rumors, the platform will choose strict supervision as a stakeholder.

## 5. Model testing and sensitivity analysis

In this paper, according to the parameter assignment method in Ref. [13–17], and considering the interaction relationship between parameters, the parameters are assigned, $a=\text{0}.\text{3},{\;C}_{P}=\text{1}\text{0},{\;R}_{P}=\text{4}\text{0},{\;P}_{\text{3}}=\text{1}\text{0},{\;M}_{\text{1}}=\text{3}\text{0},{\;M}_{\text{2}}=\text{4}\text{0},{\;I}_{\text{1}}=\text{1}\text{3},{\;R}_{\text{1}}=\text{1}\text{5},{\;R}_{\text{2}}=\text{2}\text{2},{\;C}_{\text{1}}=\text{2}\text{5},{\;C}_{\text{2}}=\text{4}\text{0},{\;S}_{\text{1}}=\text{3}\text{5},{\;S}_{\text{2}}=\text{2}\text{0}.$ It should be noted that the specific threshold values identified in the sensitivity analysis (e.g., a > 0.5, S₂ ≥ 24, P₃ < 12, R_p_ > 45) are derived from the current parameter configuration, which is theoretically calibrated based on existing literature. These values should be interpreted as relative critical points within this specific parameter space, rather than absolute empirical standards. They serve to illustrate the directional influence and relative magnitude of each parameter’s effect on system evolution, providing qualitative insights for platform strategy design rather than precise quantitative prescriptions for real-world implementation.

**Fig 3:**
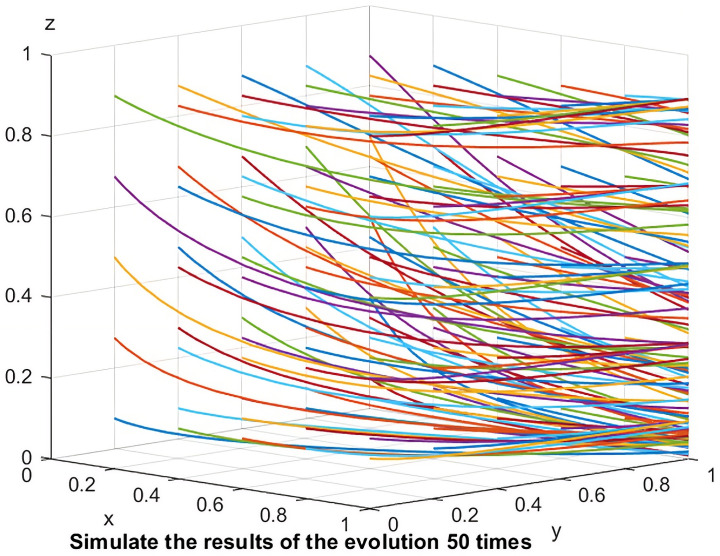
Simulate the results of the evolution 50 times.

These values are theoretically calibrated based on existing literature and academic conventions, with the core purpose of verifying the evolutionary trend of the tripartite game model and the influence mechanism of core parameters on platform strategy selection, the simulation results are shown in Fig 3.

From Fig 3, it can be seen that the stable strategy for the evolution of the game system under the experimental conditions is $E_{\text{7}}(\text{1},\text{1},\text{0})$ which means that the rumor spreader spreads the rumor, the clarifier restores the truth, and the platform chooses loose regulation for the sake of heat traffic returns. This aligns with the actual information distribution scenario, suggesting that the platform was not involved in the information process review and that, in practice, the platform still depends on the clarifier to bring the truth back to light. Clarifiers may hold off on speaking, which could lead to the widespread propagation of misleading information and have a negative social impact. The platform must assume regulatory accountability in order to preserve a stable network architecture and a safe online community. With the implementation of the clean-up campaign and rumor management, the platform gradually plays a role in pre reviewing comments and implementing measures such as account suspension and ban on verifying rumors, which has led to the transition of the evolutionary stability strategy to $E_{\text{8}}(\text{1},\text{1},\text{1})$.

**Fig 4:**
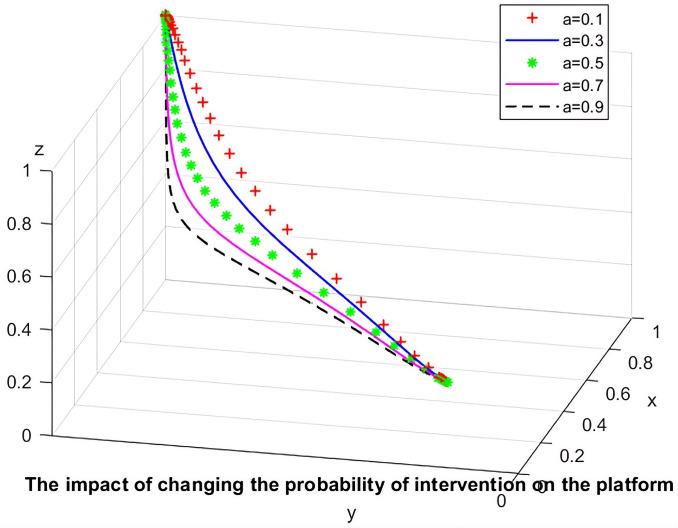
The impact of changing the probability of intervention on the platform.

**Fig 5:**
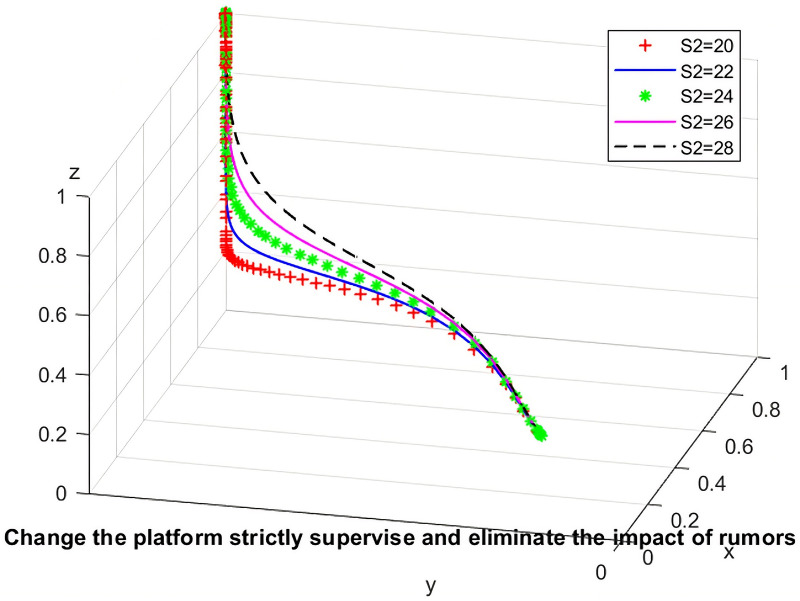
Change the platform strictly supervise and eliminate the impact of rumors.

**Fig 6:**
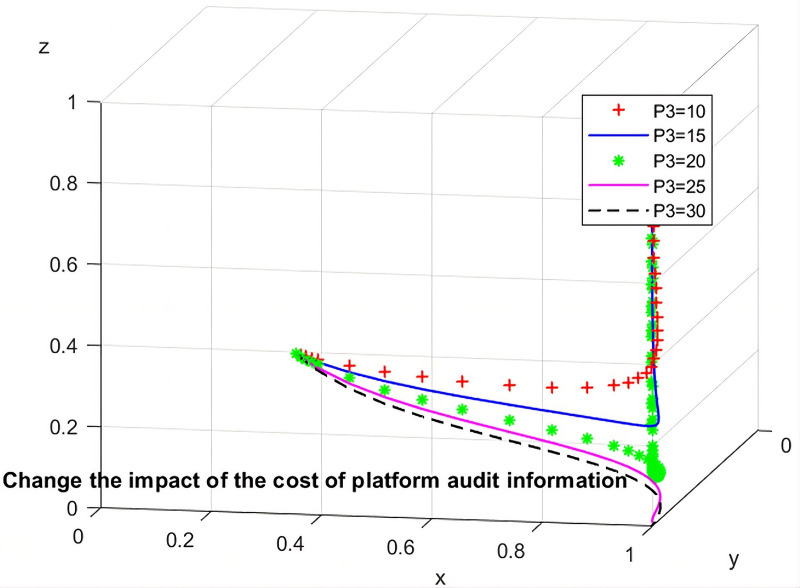
Change the impact of the cost of platform audit information.

**Fig 7:**
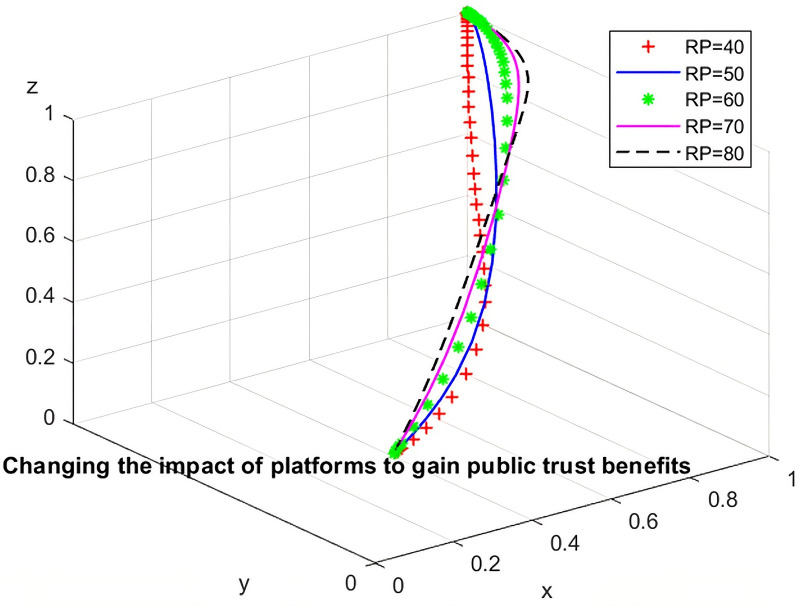
Changing the impact of platforms to gain public trust benefits.

Based on the platform preintervention affecting factors, a simulation study will be performed in the upcoming paper. We will explore, through sensitivity analysis, how the choice of stabilization strategies for a platform is affected by factors such as platform pre-intervention probability, platform audit information cost, platform rumor elimination and maintenance of network order benefits, and public trust benefits gained from strict platform supervision. Design five value schemes for each of the four parameters $\;a,\;S_{\text{2}},\;P_{\text{3}}$, and Rp to study the impact on platform strategy selection. The simulation results are shown in Figs 4–7.

Fig 4 illustrates the sensitivity of the platform’s strategy to changes in the pre-intervention probability (a). As the value of a increases, the platform’s evolutionary trajectory shifts from converging to lax supervision (z = 0) to converging to strict supervision (z = 1). Notably, when a exceeds a critical range around 0.5, the probability of the platform choosing strict supervision rises rapidly and converges to 1. This suggests that, under the given parameter configuration, a medium-to-high intervention probability is a key prerequisite for steering the system toward the ideal equilibrium E₈(1,1,1). The specific threshold value, however, may vary with different baseline parameter settings.

Fig 5 reveals a threshold effect in the benefit of strict supervision (S₂). When S₂ is below a critical value (approximately 24 in our simulation), the platform’s strategy converges to lax supervision. Once S₂ exceeds this relative threshold, the platform stably evolves toward strict supervision. This suggests that, under the current parameter configuration, the comprehensive benefit of strict supervision needs to reach a sufficiently high level—roughly double the baseline value of S₁ to motivate the platform to abandon short-term traffic gains in favor of long-term reputation building.

At present, the regulatory authorities of the platform do not have strong efforts in information review. It is necessary to incentivize clarifiers to speak up accurately and contact relevant government departments in a timely manner to respond to netizens’ doubts, eliminate the rumors caused by unclear event summaries, increase information transparency through strict regulatory review, and maintain a healthy public opinion environment.

Fig 6 illustrates the constraining effect of audit cost (P₃) on platform supervision. There exists a cost tolerance threshold: when P₃ is below approximately 12, the platform maintains a strong incentive for strict supervision; once P₃ exceeds this relative threshold, the platform progressively shifts toward lax supervision as costs become prohibitive. This implies that, within our simulation framework, keeping audit costs below a certain level—roughly comparable to the baseline traffic benefit—is essential for sustaining strict regulatory engagement.

Fig 7 demonstrates that the public trust benefit (R_p_) serves as a key driver for strict supervision. A motivational threshold is observed around 45: when R_p_ falls below this value, the platform’s strategy is sensitive to initial conditions and may converge to either equilibrium; when R_p_ exceeds this relative threshold, the platform robustly evolves toward strict supervision. This indicates that, under our parameterization, sufficiently high long-term reputation gains—substantially exceeding short-term traffic benefits—can effectively outweigh the costs of strict regulation.

In conclusion, it is discovered that the likelihood of preintervention by the platform has the greatest impact on whether a platform selects rigorous regulation. It is more advantageous to maintain a good network order, keep the online space fresh, and protect citizens’ legitimate rights and interests if the platform diligently carries out its primary duty, takes the initiative to grasp the information content, and then reviews and inspects the information published on the platform. This finding identifies platform pre-intervention probability a as a key factor that guides the system toward the ideal equilibrium $E_{\text{8}}$(1,1,1), and offers supportive evidence for designing proactive platform intervention strategies. These identified key thresholds and turning points of core parameters provide clear quantitative reference for platforms to formulate targeted intervention strategies and balance the cost-benefit of rumor governance in practice. This study uses univariate sensitivity analysis. The thresholds depend on baseline parameters, and the conclusions need to be interpreted cautiously.

## 6. Instance verification and analysis

This study takes the “Hengshan bus turning without braking” rumor event as a case study to verify the consistency between the evolutionary stable states (1,1,0) and (1,1,1) derived from the model and the evolution pattern of real online public opinion events.

### 6.1. Overview of the event

On January 2, 2023, a Douyin user @ Suifeng released a short video saying that the bus on the Hengshan Mountain in Hunan did not step on the brake too fast when turning, and regarded tourists’ lives as nothing. The video was mixed with a lot of dissatisfied words such as “you are not responsible for our lives”, “I’m afraid to get off”, and “Don’t joke with the driver”. Subsequently, the video was reposted by media outlets including The Beijing News and Henan Television, sparking widespread attention. It garnered over 174,000 likes and more than 31,000 comments, reflecting public concern over issues involving life safety. Only after 48 hours did Douyin’s official account @DouyinFactCheck release a clarification video, explaining that the driver had safely navigated the curve using the vehicle’s inertia and urging netizens to stop spreading misinformation.

Connecting the Case to the Model. This real-world incident serves as an illustrative example of the dynamic interactions captured by our model. The initial phase, where the platform did not officially intervene, corresponds to a scenario with low platform supervision intensity (low *z*). The subsequent official debunking by the platform represents a shift to a high-supervision strategy (high *z*). We use the collected data to parameterize the initial state of the SICR compartments and to qualitatively observe how changes in platform behavior align with the shifts between evolutionary stable states predicted by our model (specifically, from $E_{\text{7}}$ towards $E_{\text{8}}$).

### 6.2. Trends in propagation without the intervention of the platform

#### 6.2.1. Data acquisition and processing.

To analyze the rumor propagation process on platforms without intervention, we selected the short-video platform Douyin for event data collection. By designing a Python crawler program, we gathered comment data from a video titled “Hunan Hengshan Driver Scares Passengers by Not Braking” posted by the Henan Broadcasting account. This rumor video garnered over 31,000 comments, revealing the “traffic-driven” nature of rumor spreaders. While some netizens in the comment section attempted to clarify the facts, their efforts proved limited. This reflects the challenges faced by fact-checkers in the absence of platform coordination (i.e., lacking a positive *aC_p_* effect).

Python web scraping technology was employed, with IP rotation and request interval control ensuring data integrity and legitimacy. Collected data underwent deduplication and invalid content filtering, following this process: (1) Remove duplicate comments from the same user; (2) Eliminate meaningless interjections, pure emojis, and spam advertisements; (3) Filtering event-irrelevant content; (4) Applying keyword matching (e.g., “Hengshan Bus,” “brakes”) to retain event-related comments. From the filtered valid comments, 7,152 representative comments were selected via systematic sampling for in-depth sentiment analysis, ensuring balanced coverage across time periods and emotional orientations. Sentiment analysis employed SnowNLP for initial classification, followed by manual review of 1,000 randomly sampled comments. This achieved 92.4% agreement, validating the accuracy of sentiment categorization. Analysis revealed: 4,412 negative/critical comments were classified as Rumor Spreaders (I), primarily expressing accusations and concerns regarding passenger safety. 2,669 positive comments were classified as clarifiers (C), advising others to view the video rationally and pointing out that the clips contained edited segments taken out of context. 71 neutral or off-topic comments were categorized as restorative (R), representing users who had lost interest or exited the discussion.

Data clarification: The 174,000 likes and 31,000 comments mentioned in Section 6.1 refer exclusively to the original rumor video. This section’s core analysis is based on the 31,000 comments under that rumor video. The 437 comments under the official debunking video are analyzed separately in Section 6.3.1. These two datasets originate from different videos and are not directly comparable, but together they present the complete evolution of the incident. All data collection and analysis strictly complied with Douyin’s User Agreement and Developer Policy.

#### 6.2.2. Case simulation.

Based on the sentiment analysis results from the 7,152 sampled comments, we calibrated the initial state variables of the SICR model. In our classification, the 71 neutral/off-topic comments represent users who had already exited the discussion at the initial stage, classified as recovered (R). The remaining 7,081 sampled comments represent engaged users, which in the SICR framework serve as the pool from which rumor spreaders (I) and clarifiers (C) emerge. Therefore, we set: S (susceptible) = 7,081 (engaged users who may later adopt either stance); I (rumor spreaders) = 4,412 (negative comments identified in the sample); C (clarifiers) = 2,669 (positive comments identified in the sample); R (recovered) = 71 (users who had already lost interest).

**Fig 8:**
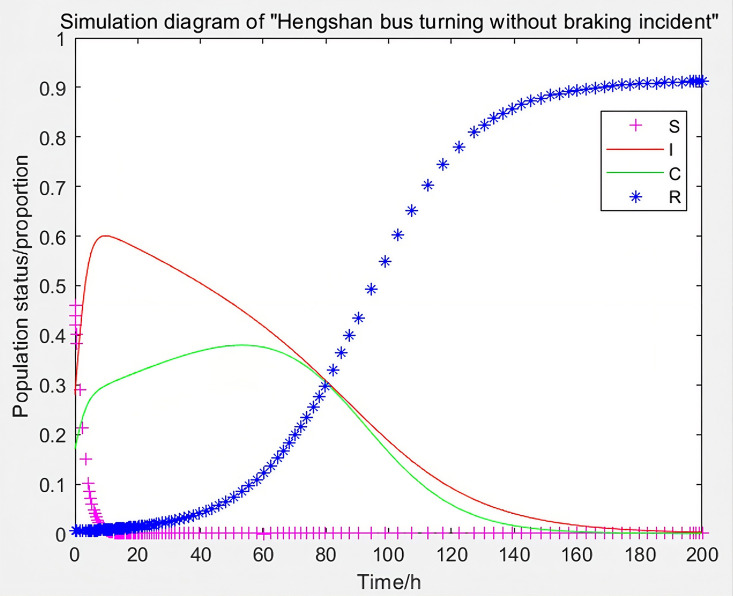
Simulation diagram of “Hengshan bus turning without braking incident”.

These values reflect the initial distribution of user states at the moment of data collection. The simulation results using these parameters are shown in Fig 8.

As illustrated in Fig 8, in the absence of platform intervention, the number of rumor spreaders (I) gradually declines over time as clarifiers (C) attempt to restore factual accuracy. However, this decline is slow, and a residual number of rumor spreaders persist even as the event evolves. Meanwhile, most engaged users gradually transition to the recovered state (R), losing interest in the discussion without the rumor being fully resolved. This pattern aligns with the characteristics of equilibrium E₇(1,1,0), where rumor spreaders and clarifiers coexist, but the platform maintains loose supervision.

### 6.3. Trends in communication under platform interventions

#### 6.3.1. Data acquisition and processing.

To examine rumor dynamics under active platform intervention, we collected comments from the official debunking video titled “Hunan Hengshan drivers don’t step on the brakes when going down the hill and going around the corner,” posted by the Blue V certified Douyin account @Douyin Rumor Refutation on January 4. Public sentiment rapidly subsided after the platform released this debunking video, validating the effectiveness of the platform’s “collaborative clarification” feature. A small number of rumor spreaders continued disseminating information, demonstrating their pursuit of long-term trust benefits (*M₂*).

After cleaning, 437 valid comments were obtained from the debunking video. Sentiment analysis revealed: 170 negative comments, mostly expressing residual skepticism or discontent; 131 positive comments, generally acknowledging the clarification and urging rational discussion; The remaining comments were neutral or off-topic.

#### 6.3.2. Case simulation.

**Fig 9:**
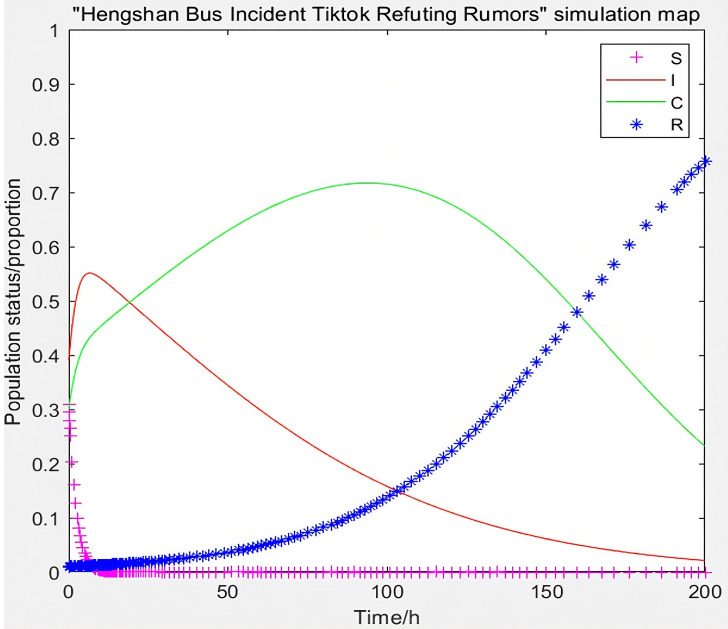
“Hengshan Bus Incident TikTok Refuting Rumors” simulation map.

Based on these results, we calibrated the SICR initial state variables for the platform intervention scenario: S (susceptible) = 135 (users who remained undecided after the debunking); I (rumor spreaders) = 170 (users continuing to spread skepticism); C (clarifiers) = 131 (users actively supporting the clarification); R (recovered) = 4 (users who had already lost interest).The simulation results are shown in Fig 9.

From Fig 9, it can be seen that following the platform’s release of the debunking video, the number of clarifiers (C) rapidly increased, effectively restoring factual accuracy and providing reasonable explanations. This not only facilitated the dissemination of truthful information but also helped eliminate doubts among netizens and maintain a healthy online discussion environment. The results demonstrate that proactive platform intervention—through strict supervision and timely dissemination of factual information—can effectively accelerate rumor suppression.

### 6.4. Analysis of results

The trends observed in the “Hengshan Bus” incident align with the evolutionary stable states derived from our model. Fig 8, depicting the scenario without active platform intervention, exhibits dynamics consistent with the conditions leading to stable point $E_{\text{7}}(\text{1},\text{1},\text{0})$ (spread, clarify, loose supervision). In this state, the burden of truth-seeking falls primarily on clarifiers, and rumor suppression is slow. In contrast, Fig 9, following the platform’s official debunking, reflects a shift towards conditions favoring stable point $E_{\text{8}}(\text{1},\text{1},\text{1})$ (spread, clarify, strict supervision). The rapid increase in clarifiers (C) and the quicker decline of rumor spreaders (I) demonstrate the accelerating effect of platform intervention on rumor suppression. This empirical observation is consistent with a key finding from our sensitivity analysis: that increasing the platform’s pre-intervention probability (parameter a) and the benefits of strict supervision ($S_{\text{2}}$) can drive the system from the inefficient equilibrium $E_{\text{7}}\;$ towards the ideal equilibrium $E_{\text{8}}$.

Therefore, this case study strongly suggests that proactive platform regulation is more effective than passive reliance on clarifiers alone. The dynamic evolution of the incident from the early *E₇* scenario to the late *E₈* scenario also provides direct empirical evidence for these two ESS matching actual online public opinion dissemination. To foster a healthy online ecosystem, platforms should proactively assume the role of critical regulatory nodes, actively disseminate accurate information, and collaborate with authoritative information agencies to spread factual truths.

To compare the trend consistency between model simulations and observed event outcomes: In the no-intervention scenario, the model predicts an 82% reduction in rumor spreaders (from 4,412 to approximately 800) and a 31% increase in clarifiers (from 2,669 to approximately 3,500). This corresponds to the actual event observation that, without platform intervention, rumors continued to circulate and spontaneous clarification remained limited. In the platform intervention scenario, the model predicts an 88% reduction in rumor spreaders (from 170 to approximately 20) and a 137% increase in clarifiers (from 131 to approximately 310), reflecting the rapid rumor suppression observed after the official debunking video was released.

Although time-series sentiment data was unavailable for rigorous RMSE calculation, this end-state comparison provides preliminary quantitative support for the model’s validity. The case validation only reflects trend consistency rather than strict quantitative prediction. Since model initialization and validation both use comment data from the same event, there exists a potential risk of circularity. The conclusion is limited to Chinese short-video platforms.

## 7. Summary and outlook

This paper investigates the behavioral strategies of participating entities in Chinese short video rumor propagation and examines control mechanisms through a combined approach combining evolutionary game theory with the SICR information dissemination model. By analyzing strategic interactions among platforms, rumor spreaders, and information clarifiers, this study evaluates the effect of various evolutionary stability strategies on curbing rumor spread in short video contexts. Numerical simulations and model validation were conducted using MATLAB, supported by empirical data from the “Hengshan Bus” incident. The results demonstrate that stringent platform oversight and timely rumor refutation are critical factors in mitigating rumor dissemination on short video platforms.

Regarding practical applications, the findings can assist short-video platforms in optimizing targeted rumor governance strategies based on predictive simulations, with targeted insights and operable measures formulated around the core sensitive factors from sensitivity analysis. The key targeted insight is that improving the platform’s pre-intervention probability (a) and balancing the cost-benefit of strict supervision are the core of effective short-video rumor governance. Corresponding actionable measures are proposed: (1) Build a high-risk information early warning system to raise the pre-intervention probability; (2) Link strict supervision with brand reputation to amplify the comprehensive benefits of $S_{\text{2}}$ and $R_{P}$; (3) Empower content review with AI technology to reduce the audit cost; (4) Establish a platform-clarifier collaboration mechanism to leverage the a$C_{P}$ synergy and lower clarifiers’ debunking costs. These targeted measures effectively guide the tripartite game system to evolve toward the ideal equilibrium $E_{\text{8}}$ (1,1,1), and provide clear practical guidance for constructing a healthy short-video information ecosystem and optimizing digital misinformation governance.

### 7.1. Theoretical contributions and innovations

Using evolutionary game theory and the SICR information diffusion model, this study identifies four theoretical and methodological insights relevant to rumor governance on short video platforms. These results help advance current research and provide a basis for future work.

First, it revises and enriches the framework of rumor governance by taking the platform as an independent bounded rational strategic actor. Breaking the traditional research paradigm that regards the platform as an exogenous regulatory variable or neutral information conduit, this study clarifies the dual attribute of the platform as a “commercial entity and governance subject” and its strategic choice dilemma, enriching the theoretical connotation of multi-subject co-governance of digital misinformation in the short video era.

Second, it proposes a combined modeling method of SICR information diffusion and tripartite evolutionary game. By combining the game strategies of the three parties (platform, rumor spreader, clarifier) with the state variables (S/I/C/R) of the SICR model, this study realizes the organic combination of two separate research paradigms (information propagation dynamics and multi-subject game behavior), expanding the application boundary of both the SICR model and evolutionary game theory in rumor governance research.

Third, it clarifies the important influence path and quantitative threshold of platform intervention on the evolutionary equilibrium of rumor governance. Through the stability analysis of the Jacobian matrix and sensitivity analysis of core parameters, this study identifies the core driving factors and constraint factors for the system to evolve toward the ideal equilibrium, and quantifies the threshold conditions for the platform to choose strict supervision strategies, providing a new theoretical perspective for the optimization of short video platform regulatory strategies.

Fourth, it provides supportive empirical evidence of theoretical model validation based on real short video platform data. Taking the typical “Hengshan Bus” incident as a case, this study crawls real comment data through Python for sentiment analysis and parameter calibration, and verifies the applicability of the theoretical model in different platform intervention intensity scenarios. This research design makes up for the deficiency of pure numerical simulation in existing studies, and significantly enhances the external validity and practical value of the theoretical model.

### 7.2. Limitations

First, the empirical data originates solely from a single short-video platform, resulting in a relatively biased data source. Furthermore, due to platform API limitations, we are unable to obtain time-series sentiment data, precluding quantitative validation of model predictions through actual public opinion trajectories. Consequently, the case study validation in Section 6 primarily relies on qualitative comparisons between simulation results and observable event characteristics, rather than strict quantitative metrics such as RMSE or R².

Second, the model incorporates several simplifying assumptions: First, assuming a uniform mixture within the SICR framework may oversimplify real-world social network structures. Second, it assumes actors do not distinguish between core influencers and ordinary users, nor does it differentiate between official institutions and grassroots groups among rumor clarifiers. Parameters are treated as static influences, failing to capture dynamic changes. While these simplifications help distill the core “feature-parameter-strategy” mechanism, they also point the way for future optimization.

Third, the sensitivity analysis employed in this study is univariate, which can only reveal the independent influence of single parameters on system evolution. This approach cannot quantitatively capture combining interaction effects between multiple parameters—such as the interaction between intervention probability a and audit cost P₃, or the synergistic effect between strict supervision benefit S₂ and public trust benefit R_p_. Consequently, the nonlinear influence mechanisms of multi-parameter variations on rumor governance effectiveness remain underexplored. This model also fails to account for the potential psychological impact of user herd behavior. In the current payoff structure, the platform strategy z does not directly affect the replicator dynamics of rumor spreaders, which constitutes a key structural limitation of the model.

Future research should integrate data from multiple video platforms, incorporating network topology, user sentiment dynamics, cross-platform spillover effects, and behavioral heterogeneity into the model to enhance its realism and predictive capability. Collecting longitudinal comment data will enable time-series validation, allowing for precise assessment of the model’s predictive accuracy.

### 7.3. Future research directions

Future research may extend this model by combining multi-platform interactions and designing deep learning-based emotion evolution modules. Empirical validation using more diverse datasets will enhance the generalizability of research conclusions. Specifically, to overcome the limitations of univariate sensitivity analysis, subsequent studies should incorporate multivariate sensitivity analysis methods such as Sobol indices. This methodology enables quantitative decomposition of first-order main effects, second-order interaction effects, and total effects for core parameters (e.g., a, P₃, S₂, R_p_). It systematically reveals the combined interaction mechanisms among multiple parameters and their combined influence on the evolution of the three-party game system toward an ideal equilibrium. Such analysis provides more comprehensive quantitative guidance for optimizing platform intervention strategies and enhancing rumor governance effectiveness.

## Supporting information

S1 Text Source code of computational simulation. (DOCX)

S2 Dataset Processed research raw data. (ZIP)
