# Supplementary material for: Governing digital misinformation: A computational analysis of platform intervention using a three-party evolutionary game model
Source: PLoS One. 2026 Jul 17;21(7):e0351207. doi: 10.1371/journal.pone.0351207 (PMC13379142; doi:10.1371/journal.pone.0351207)
Supplement: S1 Text — (DOCX) [file pone.0351207.s001.docx]

**Collect data**

import pandas as pd

import re

import jieba

from collections import Counter

class CommentDataCleaner:

"""

Douyin comment data cleaning class

Corresponds to the data cleaning process in Section 6.2.1 of the paper

"""

def __init__(self, min_length=5, stopwords_file=None):

"""

Initialize cleaner

:param min_length: minimum valid comment length (characters)

:param stopwords_file: stopwords file path (optional)

"""

self.min_length = min_length

self.stopwords = self._load_stopwords(stopwords_file) if stopwords_file else set()

# Event-related keywords (set based on the "Hengshan Bus" incident)

self.event_keywords = [

'Hengshan', 'bus', 'driver', 'brake', 'turn', 'no braking',

'life', 'safety', 'tourist', 'drive', 'steering wheel', 'inertia'

]

def _load_stopwords(self, filepath):

"""Load stopwords"""

with open(filepath, 'r', encoding='utf-8') as f:

return set([line.strip() for line in f.readlines()])

def remove_duplicates(self, df, id_col='user_id', content_col='content'):

"""

Step 1: Remove duplicate comments from the same user

Corresponds to paper: "Remove duplicate comments from the same user"

"""

before = len(df)

# Deduplicate by user ID, keep the earliest comment

df_cleaned = df.drop_duplicates(subset=[id_col, content_col], keep='first')

after = len(df_cleaned)

print(f"Deduplication: {before} → {after}, removed {before-after} duplicate comments")

return df_cleaned

def filter_meaningless(self, df, content_col='content'):

"""

Step 2: Eliminate meaningless comments

Corresponds to paper: "Eliminate meaningless comments under 5 characters,

pure emoji comments, spam advertisements"

Meaningless comment judgment rules:

1. Length less than min_length

2. Pure emoji (only emojis or special symbols)

3. Pure numbers or pure punctuation

4. Ad keyword matching

"""

def is_meaningful(text):

if not isinstance(text, str):

return False

text = text.strip()

# Rule 1: Length filter

if len(text) < self.min_length:

return False

# Rule 2: Pure emoji filter (emoji range)

emoji_pattern = re.compile(

"[\U00010000-\U0010ffff\u2600-\u26FF\u2700-\u27BF]",

flags=re.UNICODE

)

if emoji_pattern.sub('', text).strip() == '':

return False

# Rule 3: Pure numbers/punctuation filter

if re.match(r'^[\d\s\.,!?;:，。！？；：]+$', text):

return False

# Rule 4: Ad keyword filter

ad_keywords = ['add WeChat', 'private message', 'WeChat', 'official account', 'click link', 'free']

for kw in ad_keywords:

if kw in text:

return False

return True

before = len(df)

df_cleaned = df[df[content_col].apply(is_meaningful)].copy()

after = len(df_cleaned)

print(f"Meaningless comment filtering: {before} → {after}, removed {before-after} comments")

return df_cleaned

def filter_by_keywords(self, df, content_col='content', mode='any'):

"""

Step 3: Filter event-related comments through keyword matching

Corresponds to paper: "Filter event-related comments through keyword matching

(e.g., 'Hengshan Bus', 'brake')"

:param mode: 'any' - match any keyword; 'all' - match all keywords

"""

def contains_keyword(text):

if not isinstance(text, str):

return False

text = text.lower()

if mode == 'any':

return any(kw.lower() in text for kw in self.event_keywords)

else: # 'all'

return all(kw.lower() in text for kw in self.event_keywords)

before = len(df)

df_filtered = df[df[content_col].apply(contains_keyword)].copy()

after = len(df_filtered)

print(f"Keyword filtering: {before} → {after}, retained {after} event-related comments")

return df_filtered

def clean_pipeline(self, df, id_col='user_id', content_col='content',

remove_dupes=True, filter_meaningless=True, filter_keywords=True):

"""

Complete cleaning process

Corresponds to the three-step cleaning process in the paper

"""

print("="*50)

print("Starting data cleaning process")

print("="*50)

original_count = len(df)

# Step 1: Deduplication

if remove_dupes:

df = self.remove_duplicates(df, id_col, content_col)

# Step 2: Meaningless comment filtering

if filter_meaningless:

df = self.filter_meaningless(df, content_col)

# Step 3: Keyword filtering

if filter_keywords:

df = self.filter_by_keywords(df, content_col)

print("="*50)

print(f"Cleaning completed: {original_count} → {len(df)}")

print(f"Valid comment retention rate: {len(df)/original_count*100:.2f}%")

print("="*50)

return df

def export_cleaned_data(self, df, output_file='cleaned_comments.csv'):

"""Export cleaned data"""

df.to_csv(output_file, index=False, encoding='utf-8-sig')

print(f"Cleaned data saved to: {output_file}")

# Usage example

if __name__ == "__main__":

# Assume you already have raw comment data

# raw_data = pd.read_csv('raw_comments.csv')

# Create sample data (for testing)

sample_data = pd.DataFrame({

'user_id': ['user1', 'user1', 'user2', 'user3', 'user4', 'user5'],

'content': [

'This driver is terrifying, completely disregarding human life!', # normal comment

'This driver is terrifying, completely disregarding human life!', # duplicate comment

'😂😂😂', # pure emoji, should be filtered

'123456', # pure numbers, should be filtered

'add WeChat to see full video', # ad, should be filtered

'I think we should view the Hengshan Bus incident rationally' # normal comment

],

'create_time': ['', '', '', '', '', ''],

'like_count': [100, 50, 10, 5, 2, 30]

})

# Initialize cleaner

cleaner = CommentDataCleaner(min_length=5)

# Execute cleaning process

cleaned_df = cleaner.clean_pipeline(

sample_data,

id_col='user_id',

content_col='content',

remove_dupes=True,

filter_meaningless=True,

filter_keywords=True

)

# View results

print("\nCleaned data:")

print(cleaned_df[['user_id', 'content', 'like_count']])

**Sentiment analysis**

import pandas as pd

import numpy as np

from snownlp import SnowNLP

import random

from sklearn.metrics import confusion_matrix, classification_report, accuracy_score

import matplotlib.pyplot as plt

import seaborn as sns

class DouyinSentimentAnalyzer:

"""

Douyin comment sentiment analysis class

Corresponds to the sentiment analysis process in Section 6.2.1 of the paper

"""

def __init__(self):

self.results = None

self.manual_check_results = None

def analyze_sentiment(self, df, text_col='content', threshold=0.5):

"""

Use SnowNLP for sentiment analysis

Sentiment orientation: >0.5 positive (clarifier), <0.5 negative (rumor spreader), =0.5 neutral (recovered)

Corresponds to paper classification:

- Negative/critical comments → Rumor spreaders (I)

- Positive/clarifying comments → Information clarifiers (C)

- Neutral/unrelated comments → Recovered (R)

"""

print("Starting sentiment analysis...")

sentiments = []

scores = []

for i, text in enumerate(df[text_col]):

try:

if pd.isna(text) or not isinstance(text, str) or text.strip() == '':

sentiments.append('R') # Empty text classified as recovered

scores.append(0.5)

continue

s = SnowNLP(text)

score = s.sentiments

scores.append(score)

# Classify based on sentiment score

if score > threshold + 0.1: # Clearly positive

sentiments.append('C') # Clarifier

elif score < threshold - 0.1: # Clearly negative

sentiments.append('I') # Spreader

else: # Neutral

sentiments.append('R') # Recovered

except Exception as e:

print(f"Error processing text: {text[:20]}..., error: {e}")

sentiments.append('R')

scores.append(0.5)

# Add analysis results

df_result = df.copy()

df_result['sentiment_score'] = scores

df_result['sentiment_category'] = sentiments

# Count each category

counts = df_result['sentiment_category'].value_counts()

print("\nSentiment analysis results summary:")

print(f"Rumor spreaders (I): {counts.get('I', 0)} comments")

print(f"Information clarifiers (C): {counts.get('C', 0)} comments")

print(f"Recovered (R): {counts.get('R', 0)} comments")

print(f"Total: {len(df_result)} comments")

self.results = df_result

return df_result

def manual_validation_sample(self, df, sample_size=1000, random_seed=42):

"""

Randomly select samples for manual verification

Corresponds to paper: "Randomly select 1000 samples for manual verification"

"""

random.seed(random_seed)

# Random sampling

sample_indices = random.sample(range(len(df)), min(sample_size, len(df)))

sample_df = df.iloc[sample_indices].copy()

print(f"\nExtracted {len(sample_df)} comments for manual verification")

print("Please annotate according to the following standards:")

print("I - Rumor spreader (negative/critical comments)")

print("C - Information clarifier (positive/clarifying comments)")

print("R - Recovered (neutral/unrelated comments)")

# Create manual annotation column

sample_df['manual_category'] = ''

# For manual annotation, export to Excel for manual labeling in practice

sample_df.to_excel('manual_validation_sample.xlsx', index=False)

print("\nSample exported to 'manual_validation_sample.xlsx'")

print("Please complete manual annotation and re-import")

return sample_df

def load_manual_results(self, filepath='manual_validation_sample.xlsx'):

"""

Load manual annotation results

"""

manual_df = pd.read_excel(filepath)

self.manual_check_results = manual_df

return manual_df

def calculate_agreement(self, auto_df, manual_df,

auto_col='sentiment_category',

manual_col='manual_category'):

"""

Calculate agreement rate between automatic classification and manual classification

Corresponds to paper: "The agreement rate between the two methods reached 92.4%"

"""

# Merge data

merged = pd.merge(

auto_df[['user_id', auto_col]],

manual_df[['user_id', manual_col]],

on='user_id',

how='inner'

)

# Calculate agreement rate

agreement = (merged[auto_col] == merged[manual_col]).mean()

# Calculate confusion matrix

y_true = merged[manual_col]

y_pred = merged[auto_col]

labels = ['I', 'C', 'R']

cm = confusion_matrix(y_true, y_pred, labels=labels)

print("\n" + "="*50)

print("Manual Verification Results Analysis")

print("="*50)

print(f"Validation sample size: {len(merged)} comments")

print(f"Agreement rate between automatic and manual classification: {agreement*100:.2f}%")

print("\nClassification Report:")

print(classification_report(y_true, y_pred, labels=labels))

# Plot confusion matrix

plt.figure(figsize=(8, 6))

sns.heatmap(cm, annot=True, fmt='d', cmap='Blues',

xticklabels=labels, yticklabels=labels)

plt.title(f'Confusion Matrix (Agreement Rate: {agreement*100:.2f}%)')

plt.xlabel('Automatic Classification')

plt.ylabel('Manual Classification')

plt.tight_layout()

plt.savefig('confusion_matrix.png', dpi=300)

plt.show()

return agreement, cm

def get_sicr_initial_states(self, df, category_col='sentiment_category'):

"""

Get initial state variables for the SICR model

Corresponds to paper: Setting initial values for S/I/C/R

"""

counts = df[category_col].value_counts()

states = {

'S': len(df), # Total sample size as S (Susceptible)

'I': counts.get('I', 0), # Rumor spreaders

'C': counts.get('C', 0), # Information clarifiers

'R': counts.get('R', 0) # Recovered

}

print("\n" + "="*50)

print("SICR Model Initial States")

print("="*50)

print(f"Susceptible (S): {states['S']} people")

print(f"Rumor spreaders (I): {states['I']} people ({states['I']/states['S']*100:.1f}%)")

print(f"Information clarifiers (C): {states['C']} people ({states['C']/states['S']*100:.1f}%)")

print(f"Recovered (R): {states['R']} people ({states['R']/states['S']*100:.1f}%)")

return states

def plot_sentiment_distribution(self, df, score_col='sentiment_score'):

"""

Plot sentiment score distribution

"""

plt.figure(figsize=(10, 6))

# Plot histogram

plt.hist(df[score_col], bins=50, alpha=0.7, color='steelblue', edgecolor='black')

# Mark classification thresholds

plt.axvline(x=0.4, color='red', linestyle='--', alpha=0.7, label='Negative threshold (0.4)')

plt.axvline(x=0.6, color='green', linestyle='--', alpha=0.7, label='Positive threshold (0.6)')

plt.xlabel('Sentiment Score', fontsize=12)

plt.ylabel('Number of Comments', fontsize=12)

plt.title('Comment Sentiment Score Distribution', fontsize=14)

plt.legend()

plt.grid(True, alpha=0.3)

# Add classification labels

plt.text(0.1, plt.ylim()[1]*0.9, 'Rumor Spreaders (I)',

ha='center', fontsize=10, color='red')

plt.text(0.5, plt.ylim()[1]*0.9, 'Recovered (R)',

ha='center', fontsize=10, color='gray')

plt.text(0.9, plt.ylim()[1]*0.9, 'Information Clarifiers (C)',

ha='center', fontsize=10, color='green')

plt.tight_layout()

plt.savefig('sentiment_distribution.png', dpi=300)

plt.show()

# Usage example

if __name__ == "__main__":

# Assume you already have cleaned data

# cleaned_df = pd.read_csv('cleaned_comments.csv')

# Create sample data (for testing)

sample_comments = [

"This driver is terrifying, completely disregarding human life! Should be severely punished!",

"I hope everyone views this rationally, the video is edited, the driver was actually using inertia to navigate the curve",

"This kind of driver should have their license revoked!",

"I watched the full video, actually the driver's operation was fine",

"Oh well...",

"Support strict investigation!",

"Don't be misled everyone, the authorities have already debunked this"

]

test_df = pd.DataFrame({

'user_id': [f'user_{i}' for i in range(len(sample_comments))],

'content': sample_comments,

'like_count': np.random.randint(1, 100, len(sample_comments))

})

# Initialize sentiment analyzer

analyzer = DouyinSentimentAnalyzer()

# Execute sentiment analysis

results = analyzer.analyze_sentiment(test_df)

# Get SICR initial states

states = analyzer.get_sicr_initial_states(results)

# Plot distribution

analyzer.plot_sentiment_distribution(results)

# Display results

print("\nExample results:")

print(results[['content', 'sentiment_score', 'sentiment_category']].head(10))

**Simulation analysis**

clear; clc; close all;

%% 1. Parameter Settings

% Basic parameters

a_values = [0.3, 0.5, 0.7, 0.9]; % Different values of intervention probability a

C_P = 10; % Platform review cost

R_P = 40; % Public trust benefit

P_3 = 10; % Platform's cost of ongoing event data collection and audit rule formulation

S_1 = 35; % Platform's benefit from lax supervision

S_2 = 20; % Platform's benefit from strict supervision

M_1 = 30; % Rumor spreader's short-term traffic benefit

M_2 = 40; % Rumor spreader's long-term credibility benefit

I_1 = 13; % Rumor creation time cost

R_1 = 15; % Social recognition benefit from independent debunking

R_2 = 22; % Benefit from platform-assisted debunking

C_1 = 25; % Clarifier's cost of finding truth

C_2 = 40; % Clarifier's loss from silence

% Initial strategy probabilities

x0 = 0.5; % Rumor spreader's initial strategy (x)

y0 = 0.5; % Clarifier's initial strategy (y)

z0 = 0.5; % Platform's initial strategy (z)

% Time settings

t_span = [0 30]; % Evolution time range

%% 2. Color Settings (to distinguish different a values)

colors = [

0.2 0.4 0.8; % a=0.3 - blue

0.2 0.7 0.3; % a=0.5 - green

0.9 0.6 0.1; % a=0.7 - orange

0.8 0.2 0.2; % a=0.9 - red

];

%% 3. Create Figure

figure('Position', [100, 100, 800, 600]);

hold on;

grid on;

box on;

%% 4. Evolutionary Simulation for Different a Values

for i = 1:length(a_values)

a = a_values(i); % Current intervention probability

% Define replicator dynamics equations (corresponding to Equation 4 in the paper)

% Fz = z*(z-1)*(P3 - x*RP + S1*x - S2*x - RP*y + S1*y + a*CP*x + a*CP*y + x*y*RP - x*y*S1 - x*y*a*CP)

% ODE function definition

dy = @(t, y) [

% Fx - Replicator dynamics equation for rumor spreader (Equation 8)

y(1)*(y(1)-1)*(I_1 - M_2 - M_1*y(2) + M_2*y(2));

% Fy - Replicator dynamics equation for clarifier (Equation 12)

-y(2)*(y(2)-1)*(C_2 - C_1 - C_2*y(3) + R_2*y(3) + a*C_P*y(3) + y(1)*y(3)*C_2 - y(1)*y(3)*R_1);

% Fz - Replicator dynamics equation for platform (Equation 4)

y(3)*(y(3)-1)*(P_3 - R_P*y(1) + S_1*y(1) - S_2*y(1) - R_P*y(2) + S_1*y(2) + ...

a*C_P*y(1) + a*C_P*y(2) + y(1)*y(2)*R_P - y(1)*y(2)*S_1 - y(1)*y(2)*a*C_P)

];

% Initial conditions [x0, y0, z0]

y0_vec = [x0, y0, z0];

% Solve ODE

[T, Y] = ode45(dy, t_span, y0_vec);

% Plot platform strategy z(t) evolution trajectory

plot(T, Y(:,3), 'LineWidth', 2.5, 'Color', colors(i,:), ...

'DisplayName', sprintf('a = %.1f', a));

end

%% 5. Mark Threshold Line (a=0.5 threshold)

xline(10, '--', 'Color', [0.5 0.5 0.5], 'LineWidth', 1.2, ...

'HandleVisibility', 'off');

% Add text annotation at threshold position

text(10.5, 0.2, 'Threshold a ≈ 0.5', 'FontSize', 11, ...

'Color', [0.3 0.3 0.3], 'FontWeight', 'bold');

%% 6. Graph Formatting

xlabel('Time t', 'FontSize', 14, 'FontWeight', 'bold');

ylabel('Platform Strategy z(t)', 'FontSize', 14, 'FontWeight', 'bold');

title('Impact of Intervention Probability a on Platform Strategy Evolution', ...

'FontSize', 15, 'FontWeight', 'bold');

% Set axis ranges

xlim([0 30]);

ylim([0 1]);

% Add legend

legend('Location', 'best', 'FontSize', 12);

legend('boxoff');

% Add horizontal reference lines

yline(0, '--', 'Color', [0.7 0.7 0.7], 'LineWidth', 0.8, 'HandleVisibility', 'off');

yline(1, '--', 'Color', [0.7 0.7 0.7], 'LineWidth', 0.8, 'HandleVisibility', 'off');

% Set tick font

set(gca, 'FontSize', 12);

set(gca, 'LineWidth', 1.2);

%% 7. Add Annotation Box (corresponding to key findings in the paper)

annotation('textbox', [0.15, 0.7, 0.25, 0.15], ...

'String', {

'Key Finding:',

'• a < 0.5 → converge to z=0 (lax supervision)',

'• a > 0.5 → converge to z=1 (strict supervision)',

'• Threshold a ≈ 0.5 triggers phase transition'

}, ...

'FontSize', 10, ...

'BackgroundColor', [1 1 0.9], ...

'EdgeColor', [0.5 0.5 0.5], ...

'LineWidth', 1);

%% 8. Save Graph

saveas(gcf, 'Figure4_InterventionProbability.png');

saveas(gcf, 'Figure4_InterventionProbability.fig');

print('Figure4_InterventionProbability', '-depsc'); % Vector format

disp('Figure 4 has been generated and saved.');

;
